# Extremely condensing triplet states of DPEPO-type hosts through constitutional isomerization for high-efficiency deep-blue thermally activated delayed fluorescence diodes†
†Electronic supplementary information (ESI) available. See DOI: 10.1039/c5sc04848f


**DOI:** 10.1039/c5sc04848f

**Published:** 2016-01-14

**Authors:** Jing Zhang, Dongxue Ding, Ying Wei, Hui Xu

**Affiliations:** a Key Laboratory of Functional Inorganic Material Chemistry , Ministry of Education & School of Chemistry and Material Science , Heilongjiang University , 74 Xuefu Road , Harbin 150080 , P. R. China . Email: hxu@hlju.edu.cn ; Email: ywei@hlju.edu.cn

## Abstract

The similarity of thermally activated delayed fluorescence (TADF) dyes and their hosts as pure organic molecules makes hosts predominant in intermolecular interactions and crucial to exciton harvesting and utilization in TADF diodes. **DPEPO** is the most popular high-energy-gap blue TADF host with steric *ortho*-substituted diphenylphosphine oxide (DPPO) groups for intermolecular interaction suppression, but suffers from serious efficiency roll-off due to its weak electroactivity. On the contrary, *para*-substituted DPPO with small steric hindrance is superior in intramolecular electronic coupling. In this work, four constitutional isomers of **DPEPO** are constructed as diphenylether (**DPE**) with two diphenylphosphine oxide (DPPO) groups substituted at either the 2 or 4 position, namely **22′DPEPO** (*viz.***DPEPO**), **24DPEPO**, **24′DPEPO** and **44′DPEPO**, respectively. On the basis of separation configuration, the steric effect and electroactivity of *ortho*- and *para*-substituted DPPOs are successfully integrated in **24′DPEPO**, accompanied by remarkably reduced intermolecular interactions due to its unsymmetrical configuration. Compared to its congeners, **24′DPEPO** has a rigid structure and locally excited states similar to **22′DPEPO** for interaction suppression and improved charge mobility comparable to **44′DPEPO** for charge flux balance. Significantly, by virtue of the predominant orientation effect of *ortho*-DPPO on the T_1_ location, its T_1_ state is extremely condensed onto a single phenyl, protected from intermolecular interactions by its remaining five phenyls at its maximum extent. Consequently, **24′DPEPO** endowed its **DMAC-DPS**-based deep-blue devices with state-of-the-art performance, including high color purity with chromaticity coordinates of (0.16, 0.17), external quantum efficiency (EQE) beyond 20% and EQE roll-off as low as 32% at 1000 cd m^–2^. It is shown that the device performance of **24′DPEPO** was far beyond simple integration of those of **22′DPEPO** and **44′DPEPO**, verifying the significance of host optimization.

## Abstract 2

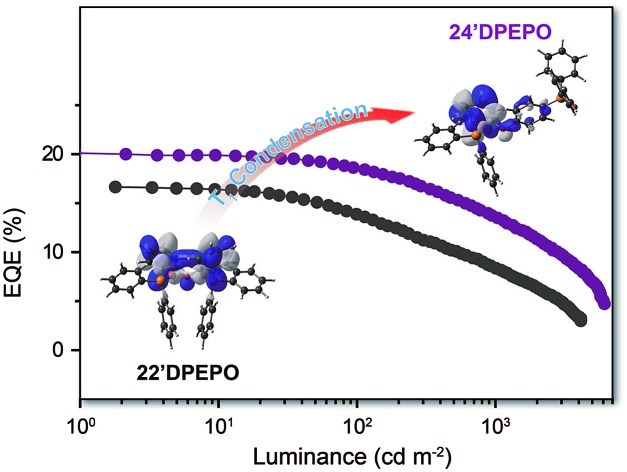
An asymmetric constitutional isomer of the thermally activated delayed fluorescence (TADF) host **DPEPO**, named **24′DPEPO**, endowed deep-blue TADF diodes with state-of-the-art performance, including external quantum efficiency beyond 20%.

## 1. Introduction

**Fig. 1:**
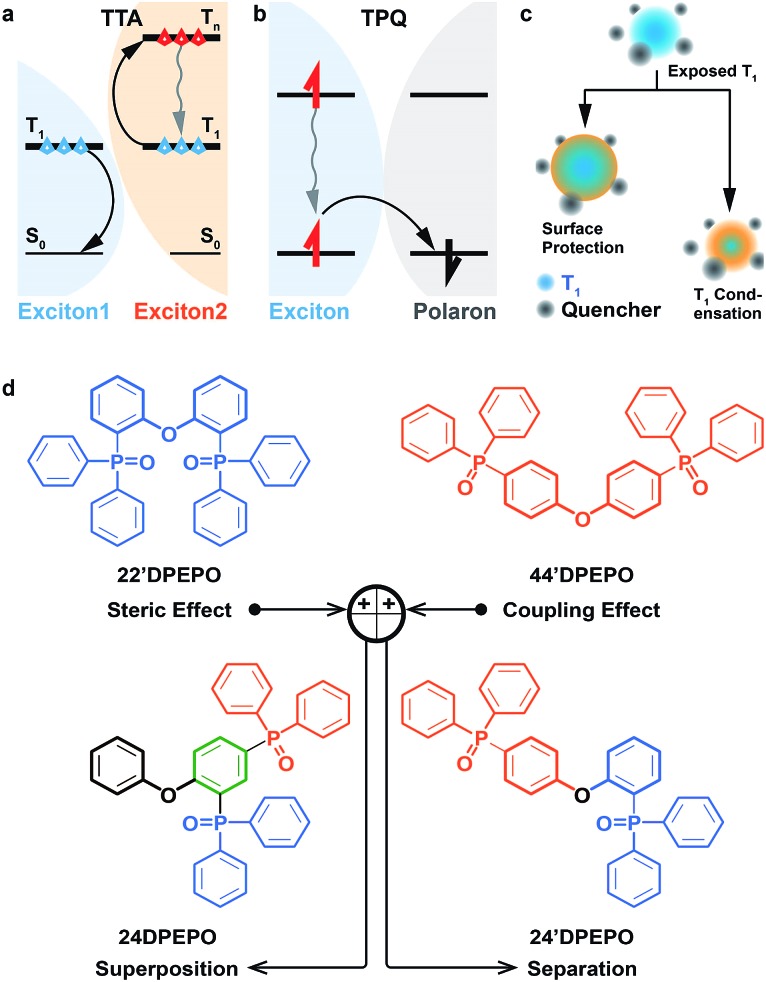
Mechanisms of triplet–triplet annihilation (TTA, a) and triplet-polaron quenching (TPQ, b), two T_1_ state protection strategies through surface modification and location condensation (c) and molecular design of **DPEPO**-type constitutional isomers ***m*DPEPO** (d).

Exciton harvesting and utilization is crucial to realize efficient organic light-emitting diodes (OLEDs).^2^ After the great success of phosphorescent heavy-metal complexes in harvesting triplet excitons through spin-orbital coupling,^3^ thermally activated delayed fluorescence (TADF) dyes have emerged in recent years, whose near-zero singlet–triplet splitting can facilitate triplet-to-singlet conversion, by virtue of reverse intersystem crossing (RISC).^4^ TADF dyes are mostly pure organic compounds featuring donor–acceptor (D–A) structures with high molecular polarity and populated with excited states characteristic of charge transfer (CT).^5^ On account of the involvement of triplet excitons in the electroluminescence process and the strong intermolecular interactions of TADF dyes, host materials are commonly adopted to dilute emitters and restrain exciton quenching in TADF diodes.^6^ However, in contrast to their phosphorescent counterparts, as the same pure organic materials, TADF dyes show molecular components and excited characteristics similar to their hosts, especially their comparable triplet lifetimes. It is rational that as it forms the majority of emissive layers (EML), the host is dominant in intermolecular interplay, including host–host and host–dopant interactions.^8^ In this case, besides the basic functions for host materials, *viz.* carrier flux balance and energy transfer, their structures and optoelectronic properties would exert great influence on collision-induced exciton quenching effects, such as triplet–triplet annihilation (TTA, Fig. 1a) and triplet-polaron quenching (TPQ, Fig. 1b).^9^ Therefore, compared to phosphorescent OLEDs, host materials in TADF diodes play a more vital role in exciton harvesting and utilization.^8^,^10^

The collision between two triplet excitons can render TTA.^11^ That is, one exciton is annihilated through transferring its energy to the other, making the latter excited to a higher level and then recovering to the first triplet (T_1_) state through internal conversion (IC) (Fig. 1a). Meanwhile, TPQ occurs during collision between an exciton and polaron, which induces the nonradiative deactivation of the former through charge exchange (Fig. 1b).^12^ In consequence, three approaches would be effective for quenching suppression: (i) embedding and/or segregating T_1_-localized moieties of host and dopant molecules to protect triplet excitons from collision quenching with surrounding modification^13^ and T_1_ state condensation,^14^ in spite of only a few reports about T_1_ condensation (Fig. 1c); (ii) reducing collision probability through shortening exciton lifetime; (iii) enhancing charge flux balance and unifying exciton recombination to decrease polaron concentration. In this case, the excited-state characteristics and carrier-transporting ability of host materials should be a determinant for their electroluminescence (EL) efficiencies.

It is known that the CT-type exciton is labile and highly sensitive to the environment.^15^ Consequently, although 100% internal quantum efficiencies were already achieved for TADF devices, most of them suffered from serious efficiency roll-off, especially for blue TADF diodes utilizing high-energy excitons. **DPEPO** is the most popular host for blue TADF diodes.4a,5e,5i,5j,^16^ Its multi-insulating structure and the steric effect of P=O groups give rise to its high T_1_ value of ∼3.0 eV for efficient energy transfer to blue TADF dyes, *e.g.* bis[4-(9,9-dimethyl-9,10-dihydroacridine)phenyl]sulfone (**DMAC-DPS**) (T_1_ = 2.90 eV)16*a* and quenching suppression.^17^ The external quantum efficiency (EQE) of deep-blue TADF devices with **DMAC-DPS** as the emitter reached ∼20%, however, this was accompanied by remarkable roll-off of more than 90% at 1000 cd m^–2^, indicating serious exciton quenching.^18^ Nevertheless, how to optimize optoelectronic properties of blue TADF host materials is really challenging, since many key issues are still unclear, especially the influences and correlations of their electrical properties and excited-state characteristics and their merits.^19^ In our previous work, it was shown that these two factors strongly depend on the functional linkage site and molecular sequence.^20^ In this sense, the isomerization of **DPEPO** may provide a feasible way to clarify the key determinants of EL performance for deep blue TADF host materials.

In this contribution, four constitutional isomers of **DPEPO** with the collective name of ***m*DPEPO** are constructed as bis(diphenylphosphoryl) diphenylether with two diphenylphosphine oxide (DPPO) groups at either the 2 or 4 position of their diphenylether (**DPE**) cores, named **22′DPEPO** (*viz.***DPEPO**), **24DPEPO**, **24′DPEPO** and **44′DPEPO**, respectively (Fig. 1d). DPPO at the 2-position indicates the strong steric effect and dominant localization effect on the T_1_ state, while DPPO at the 4-position is superior in enhancing intramolecular electronic coupling. By virtue of the asymmetrical and separated structure for **24′DPEPO**, the functions of its two kinds of DPPO groups are successfully integrated. Significantly, its T_1_ state is extremely condensed to a single phenyl, embedded and protected by its remaining five phenyls at its maximum extent. Meanwhile, compared to **22′DPEPO**, the electron mobility of **24′DPEPO** is dramatically improved by about 50 times. Consequently, its **DMAC-DPS**-based deep-blue devices realized state-of-the-art performance, including high color purity with CIE coordinates of (0.16, 0.17), an EQE beyond 20% and EQE roll-off as low as 32% at 1000 cd m^–2^. The efficiencies of **24′DPEPO**-based devices were improved by 20% in comparison to those employing **22′DPEPO** as the host; meanwhile, the roll-offs of the former were halved. It is clear that host engineering is a gateway to resolving the key issues of TADF devices in exciton harvesting and utilization.

## 2. Results and discussions

### 2.1. Design and synthesis

DPPO is an electron-withdrawing group with big steric hindrance. Its dominant function depends on its substitution positions. For instance, DPPOs at *ortho*-positions of **22′DPEPO** are superior in their steric effect; while, DPPOs in **44′DPEPO** can enhance intramolecular coupling with an electron-donating ether bridge at the *para*-position (Fig. 1c). In this case, an intermolecular interaction with **22′DPEPO** at the DPPO-substituted side can be thoroughly blocked to effectively suppress TTA. However, electronic coupling in **22′DPEPO** is remarkably weaker due to its *ortho*-substitution configuration.^17^ In contrast, sufficient electronic coupling in **44′DPEPO** can support balanced hole and electron flux in its EMLs, facilitating exciton recombination and reducing TPQ.^21^ Nonetheless, its **DPE** chromophore is completely exposed to intermolecular interactions, worsening TTA. Therefore, it is well-reasoned to combine these two kinds of DPPO substitutions in one single molecule for integrating their advantages and thereby simultaneously suppressing TTA and TPQ.

More significantly, in our recent works, it was shown that DPPO substitution can regulate T_1_ location, which should be attributed to the influence of the P=O group on electron cloud distribution.^14^,20a,^22^ In this sense, the orientation effect of DPPO on the T_1_ state would be related to its substitution position. Therefore, with asymmetrical DPPO substitutions, the T_1_ states of the molecules can be condensed into a designated location by DPPO with a dominant orientation effect. The narrow T_1_ location can amplify the protective action of surrounding groups and further reduce its probability of involvement in intermolecular interactions. Regarding **DPEPO**-type analogues, since all of their phenyls are separated by insulating linkages, it can be expected to confine their T_1_ states into a single phenyl, which is almost the minimum for conjugated compounds with respect to spatial scale.

With these considerations, two other asymmetrical isomers, namely **24DPEPO** and **24′DPEPO**, incorporating both *ortho*- and *para*-linked DPPO groups were designed. Two DPPOs in **24DPEPO** are bonded with the same phenyl, while those in **24′DPEPO** are respectively bonded with two different phenyls of **DPE**. The superposition of two DPPOs in **24DPEPO** may render the mutual acceleration or counteraction of their effects. On the contrary, by virtue of the insulating ether bridge, the two separated DPPOs of **24′DPEPO** can perform their functions independently. In consequence, it is most likely for **24′DPEPO** to integrate the merits of **22′DPEPO** and **44′DPEPO**. Simultaneously, the multi-insulating linkage establishes the similarity of ***m*DPEPO** to the maximum extent, excluding the interference from undesirable intramolecular interplay and conjugation variation, which simplifies the influencing factors of their device performance into electrical and excited-state characteristics.

***m*DPEPO** can be conveniently prepared through Pd-catalyzed phosphorylation with good yields of more than 50%. Their chemical structures were fully characterized on the basis of NMR spectra, mass spectra and elementary analysis. Among ***m*DPEPO**, the biggest steric hindrance in **22′DPEPO** increases its intramolecular tension, rendering its lowest temperature of decomposition (*T*_d_) as 322 °C; while its rigid and monosymmetrical structure results in its highest melting point (*T*_m_) of 280 °C (Fig. S1† and Table 1). On the contrary, owing to negligible steric hindrance in **44′DPEPO**, its *T*_d_ is remarkably improved to 417 °C, however, this is accompanied by a reduced *T*_m_ of 190 °C. The rotatable phenyl of **DPE** in **24DPEPO** makes its thermal properties similar to those of **44′DPEPO**. Importantly, **24′DPEPO** shows a *T*_d_ equivalent to that of **44′DEPPO** and a *T*_m_ comparable to that of **22′DPEPO**. Therefore, one *ortho*-substituted DPPO in **24′DPEPO** already revealed sufficiently strong molecular rigidity.

**Table 1:** Physical properties of ***m*DPEPO**

	**22′DPEPO**	**24DPEPO**	**24′DPEPO**	**44′DPEPO**
*λ*_abs_ ^*a*^ (nm)	227, 274, 284, 300^*b*^, 225, 283^*c*^	228, 274, 284, 300^*b*^, 225, 283^*c*^	228, 253, 274, 284, 294^*b*^, 225, 283^*c*^	228, 250, 273, 283^*b*^, 225, 278^*c*^
*λ*_Em_ ^*d*^ (nm)	312^*b*^/309, 389^*c*^	311^*b*^/316, 349, 418, 447^*c*^	310^*b*^/315, 412, 433, 461^*c*^	301^*b*^/302, 343^*c*^
S_1_/T_1_ (eV)	3.92^*e*^/2.98^*f*^, 4.82/3.64^*g*^	3.94^*e*^/2.98^*f*^, 4.73/3.53^*g*^	3.92^*e*^/2.98^*f*^, 4.76/3.62^*g*^	3.94^*e*^/2.97^*f*^, 4.75/3.53^*g*^
*T*_g_/*T*_m_/*T*_d_ (°C)	—/280/322	—/203/405	—/250/417	—/190/417
HOMO/LUMO (eV)	–6.53/–2.53^*h*^, –6.27/–0.76^*g*^	–6.51/–2.79^*h*^, –6.40/–0.97^*g*^	–6.65/–2.63^*h*^, –6.34/–0.89^*g*^	–6.65/–2.63^*h*^, –6.27/–0.93^*g*^
*E*_R_ ^*i*^ (eV)	0.2312^*j*^/0.4353^*k*^	0.7237^*j*^/0.5550^*k*^	0.2884^*j*^/1.0176^*k*^	0.1823^*j*^/0.3129^*k*^
*μ* ^*l*^ (cm^2^ V^–1^ s^–1^)	7.03 × 10^–8^^*j*^/1.40 × 10^–9^^*k*^	7.72 × 10^–9^^*j*^/1.25 × 10^–9^^*k*^	4.02 × 10^–6^^*j*^/0.99 × 10^–8^^*k*^	5.15 × 10^–6^^*j*^/1.20 × 10^–7^^*k*^
*λ*_Em_ ^*m*^ (nm)	470	470	470	470
*τ* ^*m*^ (μs)	7.2	7.4	7.5	8.6
PLQY^*m*^ (%)	85	59	89	77

^*a*^Absorption peaks.

^*b*^In CH_2_Cl_2_ (10^–6^ mol L^–1^).

^*c*^In polycrystalline powder.

^*d*^Fluorescence peaks at room temperature.

^*e*^Estimated according to the absorption edges.

^*f*^Calculated according to the 0–0 transitions of the phosphorescence spectra.

^*g*^TDDFT calculated results.

^*h*^Calculated according to the equation HOMO/LUMO = –(4.78 + onset voltage) eV.^1^

^*i*^Reorganization energy of electron.

^*j*^For electron.

^*k*^For hole.

^*l*^Electron mobility estimated by *I*–*V* characteristics of electron-only devices according to field-dependent SCLC model.^7^

^*m*^Data of vacuum-evaporated ***m*DPEPO**:**DMAC-DPS** (10% wt) films with thickness of 100 nm.

### 2.2. DFT and TDDFT simulation

For insight into the nature of the effects of DPPO substitution on electrical and excited-state characteristics of ***m*DPEPO**, DFT and TDDFT calculations were performed at the level of B3LYP/6-31+g(d,p), taking into account computational accuracy and cost.

**Fig. 2:**
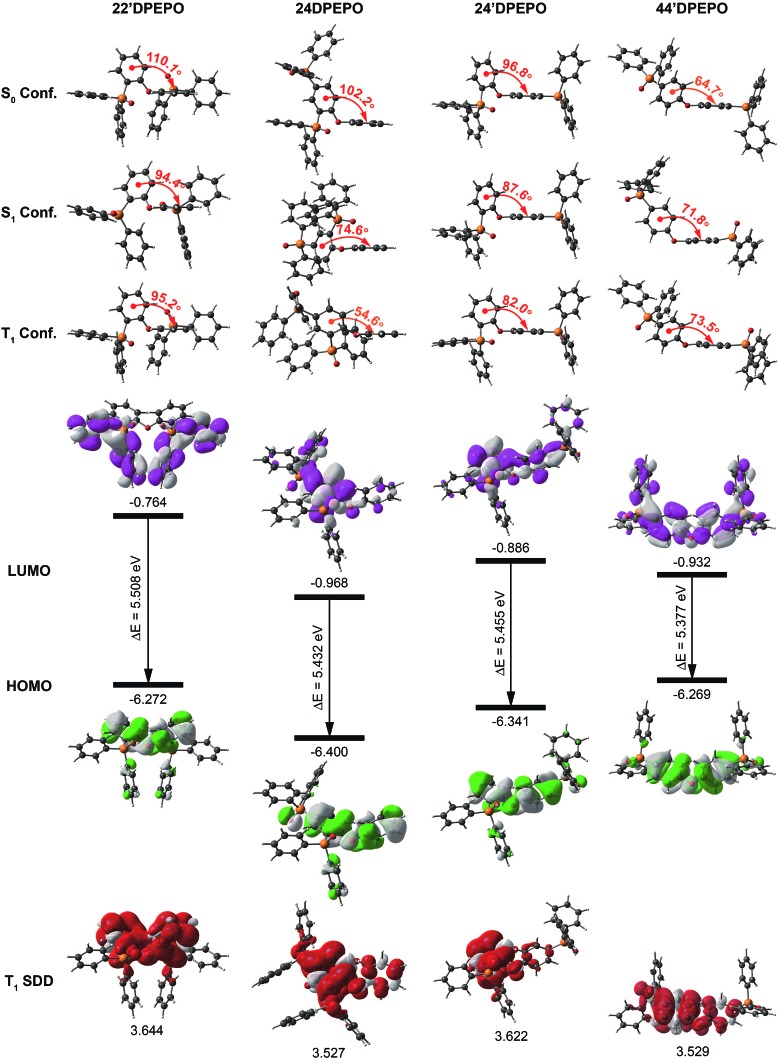
DFT and TDDFT simulations of ground, S_1_ and T_1_ excited states for ***m*DPEPO**: configurations, the LUMO and HOMO orbital distributions of ground states and the spin density distributions of T_1_ states.

The optimized molecular configurations of ***m*DPEPO** at ground (S_0_), the first singlet (S_1_) and T_1_ states are shown in Fig. 2. Ascribed to the biggest steric hindrance of its two *ortho*-substituted DPPOs, the dihedral angle of **DPE** in **22′DPEPO** at the S_0_ state is the largest among ***m*DPEPO**, which is almost preserved at its S_1_ and T_1_ states. The dihedral angles of **DPE** in **44′DPEPO** at these states are the smallest, in accord with the smaller steric hindrance of its DPPOs at the *para*-position. Compared to the ground state, its excited-state configurations are adjusted. Nevertheless, **24DPEPO** shows the largest configuration variation at excited states, which is ascribed to the rotational phenyl of its **DPE**. In this sense, both *ortho*- and *para*-DPPO substitution can reduce excited-state structural relaxation. Consequently, the molecular configurations of **24′DPEPO** at S_0_, S_1_ and T_1_ states are almost unchanged, accompanied by the dihedral angles of **DPE** close to those of **22′DPEPO**. In comparison to **24DPEPO**, the fixed molecular configurations of **24′DPEPO** and **22′DPEPO** make them superior in reducing relaxation-induced excited-energy loss, which is beneficial to improving luminescent efficiency.

The contours of the highest occupied and the lowest unoccupied molecular orbitals (HOMO and LUMO) for ***m*DPEPO** reveal the influence of DPPO substitution position on hole and electron injecting ability, respectively. Although the frontier molecular orbitals (FMO) of **22′DPEPO** are respectively localized on its **DPE** and DPPO groups, its LUMO energy level is the highest among ***m*DPEPO**, indicating the weakest electron-withdrawing ability of its DPPOs at the 2-position (Fig. 2, S2† and Table 1). The situations of **44′DPEPO** and **24DPEPO** are similar, with partially separated HOMO and LUMO. However, the superposition of DPPOs in **24DPEPO** renders the simultaneously reduced HOMO and LUMO energy levels. In contrast, **44′DPEPO** possesses a HOMO and LUMO that are almost equivalent to those of **22′DPEPO** and **24DPEPO**, respectively, causing the enhanced intramolecular electronic coupling in **44′DPEPO** and its ambipolar characteristics. As expected, through incorporating the *para*-substituted DPPO, in comparison to **22′DPEPO**, the HOMO energy level of **24′DPEPO** is preserved, while its LUMO energy level is reduced by 0.12 eV, which is similar to that of **44′DPEPO**. Therefore, it is shown that despite its overlapping HOMO and LUMO distributions, the charge injecting ability of **24′DPEPO** is mainly determined by its *para*-linked **DPE**-DPPO segment.

The calculated S_1_ and T_1_ energies of ***m*DPEPO** are almost the same as ∼4.7 and ∼3.5 eV, supporting positive energy transfer to **DMAC-DPS** with the S_1_ and T_1_ value of ∼2.9 eV (Fig. 2 and Table 1). The similar excited energy of ***m*DPEPO** is attributed to their multi-insulating structures. It is rational that on the basis of monosymmetric configurations and two equivalent DPPOs, the T_1_ states of **22′DPEPO** and **44′DPEPO** are uniformly dispersed on their **DPE** groups. In accord with our previous reports, DPPO substitution has an effect on T_1_ location regulation.20*a* The T_1_ state of **24DPEPO** is mainly contributed by the phenyl substituted with two P=O groups. Interestingly, **24′DPEPO** shows a T_1_ state that is thoroughly localized on the 2-DPPO substituted phenyl, reflecting the dominant orientation effect of *ortho*-substituted DPPO on the T_1_ state. The T_1_ state of **24′DPEPO** is extremely condensed on a single phenyl as one of the smallest conjugated units, which is embedded by its remaining five phenyls. Compared to its isomers, the exposure degree of the T_1_ state of **24′DPEPO** is doubtlessly the smallest, which can support the most effective suppression of collision-induced quenching effects.

**Fig. 3:**
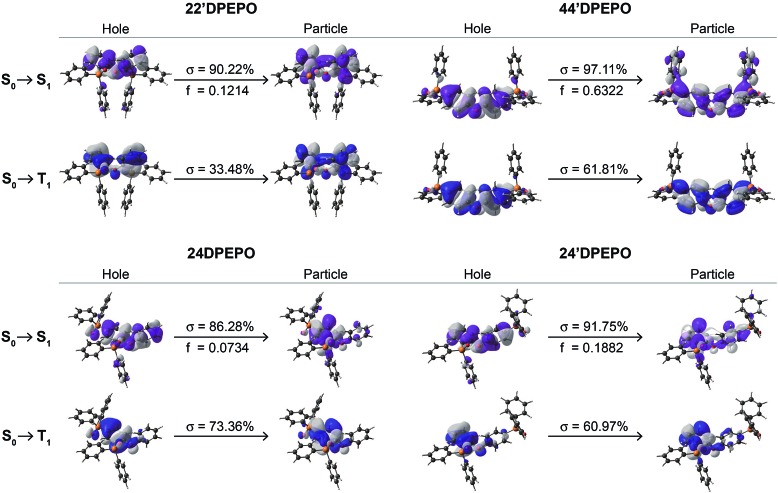
Natural transition orbitals (NTO) of the S_1_ and T_1_ states for ***m*DPEPO**. *σ* and *f* refer to the associated weight and oscillator strength, respectively.

The nature of electronic transitions for the excited states of ***m*DPEPO** was further evaluated with natural transition orbitals (NTO) of the first singlet and triplet excitations (Fig. 3 and S4†).^23^ In the cases of NTOs about S_0_ → S_1_ states for ***m*DPEPO**, “holes” are thoroughly localized on their **DPE**. However, the distributions of “particles” are various, such that for **22′DPEPO** and **24′DPEPO**, “particles” are also dispersed on their **DPE**, featuring locally excited (LE) state transition; while, for **24DPEPO**, “holes” are concentrated to one phenyl of **DPE**, and for **44′DPEPO**, “holes” are partially dispersed on DPPO groups, characteristic of hybridized local and charge transfer excited states. Nevertheless, the biggest oscillator strength (*f*) suggests the LE transition of **DPE** as the major part for **44′DPEPO**, while CT transition between two phenyls of **DPE** is dominant for **24DPEPO** with the smallest *f*. NTOs of S_0_ → T_1_ states for ***m*DPEPO** reveal an LE character with overlapped “hole” and “particle” locations. In accord with the DFT results, triplet states of **22′DPEPO** and **44′DPEPO** are dispersed on their **DPE**, while only a single phenyl of **24DPEPO** and **24′DPEPO** is involved in their triplet transitions. Regarding CT character of excitons on TADF dyes, LE-dominant excited states of hosts can provide uniform and apparently neutral matrices to restrain Coulomb interaction induced CT exciton dissociation.

It is shown that **24′DPEPO** successfully integrates the merits of *ortho*- and *para*-DPPO substitutions, including excited structural stability, singlet characteristics comparable to **22′DPEPO** and FMO energy levels close to **44′DPEPO**. In addition to significantly reinforced T_1_ state protection through an extremely condensed T_1_ location on the minimum unit phenyl, **24′DPEPO** shows superiority in quenching suppression and charge balance. In contrast, **24DPEPO** exhibits some shortages of serious excited structural relaxation and a CT-dominant S_1_ characteristic.

### 2.3. Optical properties

**Fig. 4:**
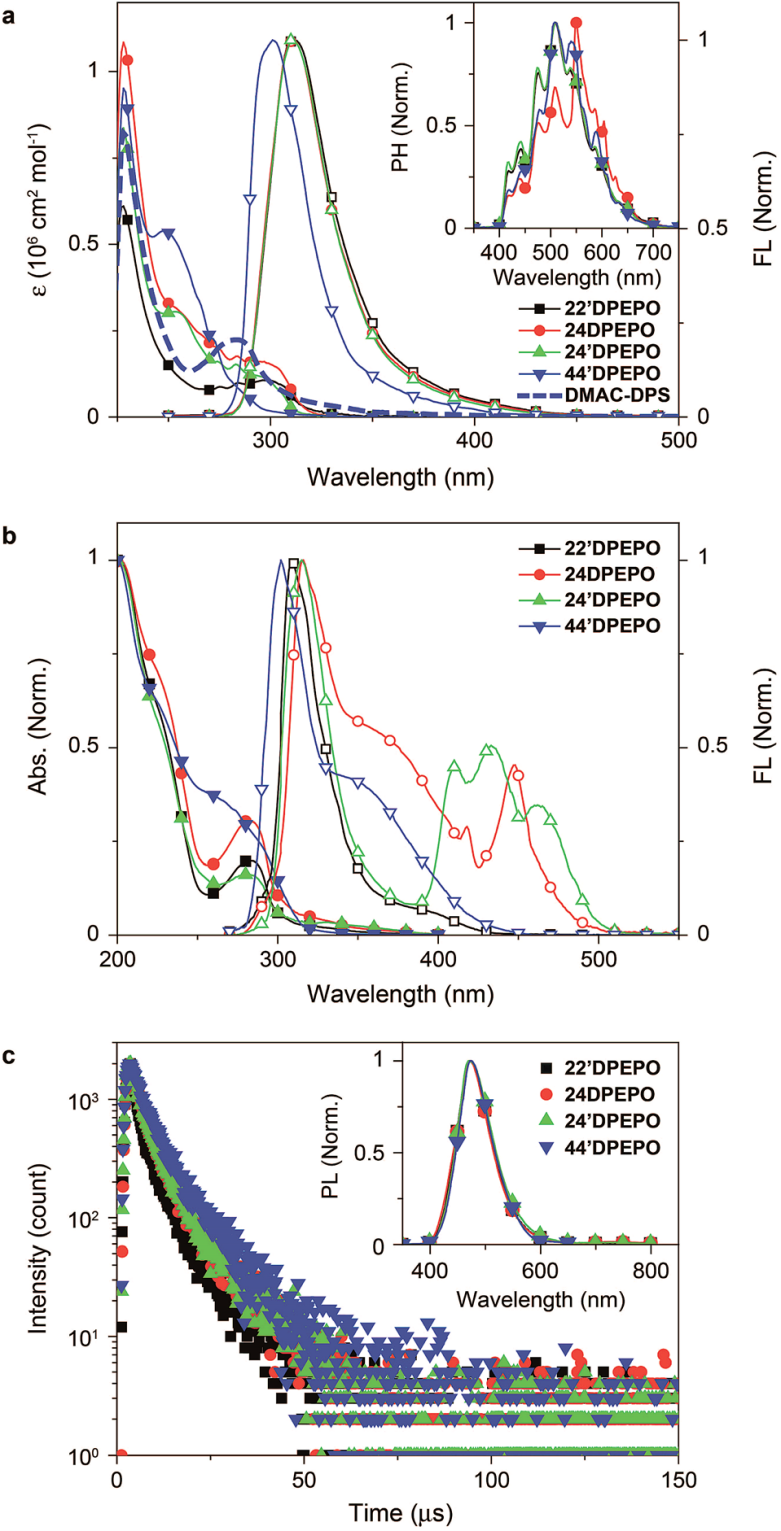
(a) Electronic absorption spectra, room-temperature emission spectra of ***m*DPEPO** in CH_2_Cl_2_ (10^–6^ M). Inset shows the time-resolved phosphorescence spectra of ***m*DPEPO** in CH_2_Cl_2_ glass at 77 K after a delay of 100 μs; (b) absorption and fluorescence (FL) spectra of polycrystalline powder for ***m*DPEPO**; (c) time decay curves and emission spectra (inset) of **DMAC-DPS**-doped ***m*DPEPO** films (10%, 100 nm) through vacuum-evaporation.

The absorption spectra of ***m*DPEPO** in dilute solutions (10^–6^ M in CH_2_Cl_2_) consist of three bands at around 230, 270 and 300 nm, corresponding to π → π* transitions of DPPO and **DPE** and the n → π* transition of **DPE**, respectively (Fig. 4a). The spectra of **22′DPEPO**, **24DPEPO** and **24′DPEPO** are almost identical, but different to that of **44′DPEPO**. As indicated by the DFT results, the smallest dihedral angle of **DPE** in **44′DPEPO** facilitates π–π interactions between the two phenyls of **DPE** and decreases p–π conjugation between the O atom and phenyls, accordingly enhancing π → π* transition and weakening n → π* transition. Nevertheless, the optical energy gaps of ***m*DPEPO** estimated by absorption edges are almost identical to 3.9 eV, consistent with TDDFT results (Table 1).

In the same way, the fluorescence (FL) spectra of **22′DPEPO**, **24DPEPO** and **24′DPEPO** in dilute solutions (10^–6^ M in CH_2_Cl_2_) are exactly the same in range and profile. Meanwhile, the FL spectrum of **44′DPEPO** reveals a blue shift of ∼10 nm. Nonetheless, there are large-range spectral overlaps between FL emissions of ***m*DPEPO** and absorption of **DMAC-DPS** from 275 to 350 nm, facilitating Förster resonance energy transfer (FRET). The low-temperature time-resolved phosphorescence (PH) spectra of ***m*DPEPO** are identical, with the same peaks and profiles, which is in accord with their similar T_1_ transition characteristics as shown by TDDFT simulation (inset of Fig. 4a). Estimated with 0 → 0 transitions, the T_1_ value of ***m*DPEPO** is equivalent to 2.98 eV, supporting the efficient triplet energy transfer to **DMAC-DPS**. The similar excited energy of these **DPEPO**-type isomers fully verifies the effectiveness of multi-insulating linkages in excited energy preservation.

The optical properties of ***m*DPEPO** in the solid state were further investigated to exclude solvent effects (Fig. 4b). The main absorption bands are preserved in the solid-state spectra, reflecting the limited intermolecular interactions. However, in contrast to the unchanged fluorescence emission of **22′DPEPO** in the solid state, **44′DPEPO** shows an additional broad aggregation-induced emission band in the long-wavelength range from 325 to 450 nm, in accord with the weak steric effect of its *para*-DPPOs. The flexible **DPE** in **24DPEPO** also facilitates aggregation, rendering a similar broad band at around 340 nm. As expected, by virtue of the strong steric effect of *ortho*-substituted DPPO in **24′DPEPO** with a separation configuration, the aggregation in the solid state is successfully suppressed, making one of its solid-state emission bands at 315 nm almost overlapped with its emission in solution. Significantly, solid-state emissions of **24′DPEPO** and **24DPEPO** contain additional distinct multi-peak bands coincident with the blue parts of their phosphorescence, whose lifetimes (*τ*) are also comparable to ∼7 μs (Fig. S3†); meanwhile, for **22′DPEPO** and **44′DPEPO**, their solid-state phosphorescence is fully quenched through nonradiative transitions due to their exposed T_1_ states. In dilute solutions, the small volumes of solvent molecules make collision with every part of solute molecules facile, resulting in serious solvent quenching effects on T_1_ states, which can be excluded in the solid state. Recently, room-temperature phosphorescence from crystalline pure organic molecules was realized on the basis of D–A systems with overlapping S_1_ and T_1_ locations.^24^ It is rational to attribute the visible room-temperature solid-state phosphorescence of **24′DPEPO** and **24DPEPO** to their extremely condensed T_1_ state, effectively protected from interaction-induced triplet quenching, which validates T_1_ condensation as a feasible and effective strategy to achieving and enhancing room-temperature phosphorescence from pure organic systems.

The host–dopant energy transfer was further investigated through steady-state and transient photoluminescence (PL) spectra of vacuum-evaporated **DMAC-DPS**-doped ***m*DPEPO** films (Fig. 4c). The emission spectra are almost identical, with the maxima at 470 nm, corresponding to the pure **DMAC-DPS**-originated emission, which indicates the same positive energy transfer from ***m*DPEPO** consistent with their equivalent S_1_ and T_1_ energy. As mentioned above, the *τ* of T_1_ states for ***m*DPEPO** and emissions of their **DMAC-DPS**-doped films is of the same order of magnitude, making ***m*DPEPO**-involved interactions dominant in triplet quenching processes. The *τ* of the **44′DPEPO**-based film is 8.6 μs, about 1 μs longer than those of the other isomer based films, revealing the effect of **44′DPEPO** with a partial CT-featured S_1_ state on stabilizing **DMAC-DPS** excitons.^8^ In spite of the CT-dominant S_1_ state, the *τ* of the **24DPEPO**-based film is much shorter due to its structural relaxation-induced exciton quenching, which is further manifested by its lowest PL quantum yield (PLQY) of only 59% (Table 1). Furthermore, it is known that a longer lifetime would worsen collision-induced exciton quenching. Therefore, in comparison to the PLQY of 77% for the **44′DPEPO**-based film, **22′DPEPO** and **24′DPEPO** endow their films with PLQYs as high as 85 and 89%, respectively.

In consequence, the optical properties of **24′DPEPO** and **22′DPEPO** are identical, originating from their similar excited-state characteristics as TDDFT simulated.

### 2.4. Electrical properties

**Fig. 5:**
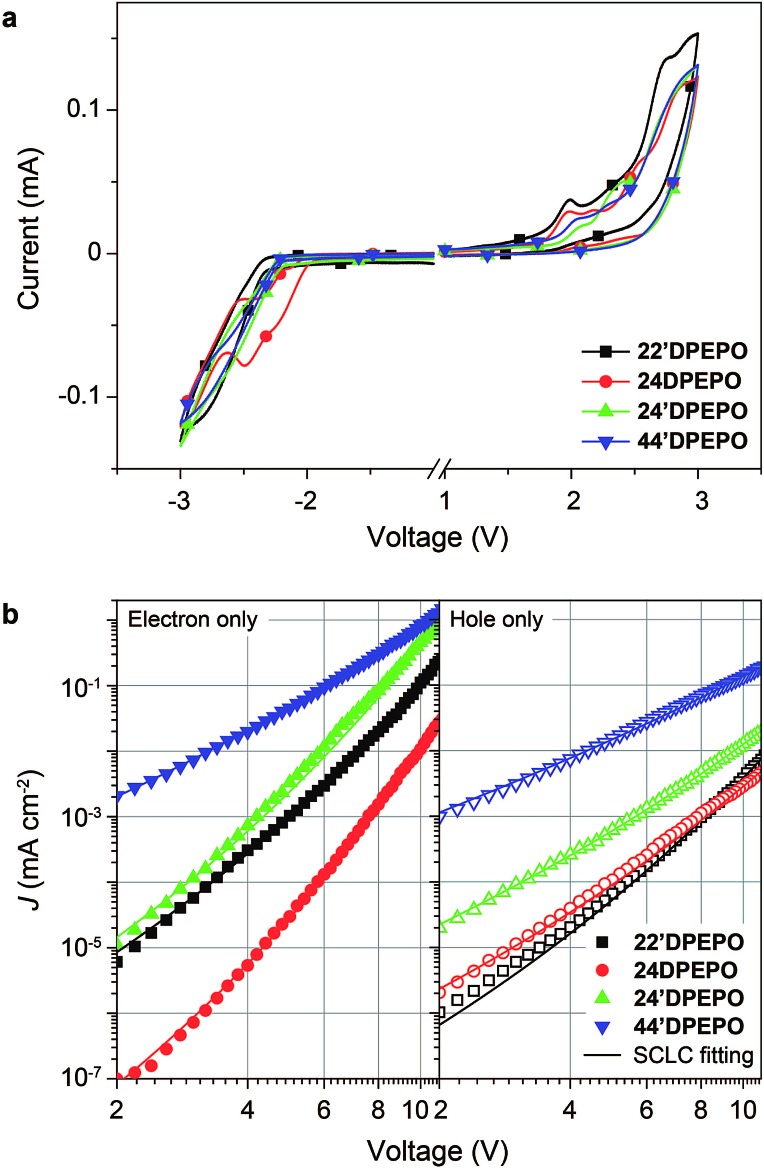
(a) Cyclic voltammograms of ***m*DPEPO** measured in tetrahydrofuran for reduction and dichloromethane for oxidation, respectively, at room temperature with tetrabutylammonium hexafluorophosphate as the electrolyte under a scanning rate of 100 mV s^–1^; (b) volt–ampere characteristics of single-carrier only transporting devices based on ***m*DPEPO** and the corresponding fitting curves according to field-dependent SCLC model.

The influence of DPPO substitution position on the HOMO and LUMO energy levels is experimentally investigated with cyclic voltammetry (CV) analysis (Fig. 5a). The oxidation voltammograms of ***m*DPEPO** consist of two irreversible peaks characteristic of **DPE** and DPPO, respectively. Estimated with onset voltages of the anodic peaks, the HOMO energy level of **24′DPEPO** is equivalent to that of **44′DPEPO** at –6.65 eV, which is 0.1 eV lower than those of **22′DPEPO** and **24DPEPO** (Table 1). On the other hand, the reduction voltammograms of **22′DPEPO**, **24′DPEPO** and **44′DPEPO** only contain single irreversible cathodic peaks, while **24′DPEPO** shows two irreversible reduction peaks attributed to **DPE** and DPPO, respectively. It is shown that the reduction curves of **24′DPEPO** and **44′DPEPO** are almost overlapped with the same onset voltages of –2.15 V, corresponding to the LUMO of –2.63 eV. The superposition of two DPPOs in **24DPEPO** further reduces its LUMO to –2.79 eV, which is in accord with the DFT results. It is clear that in comparison to **22′DPEPO**, the stronger electron-withdrawing effect of *para*-DPPO substitution endows **24′DPEPO** and **44′DPEPO** with the deeper LUMOs. Significantly, the identical FMO energy levels of **24′DPEPO** and **44′DPEPO** verify the dominant effect of *para*-DPPO in FMO energy level regulation.

The nominal single-carrier transporting devices with single-layer configurations of ITO|MoO_3_ (6 nm)|***m*DPEPO** (100 nm)|MoO_3_ (6 nm)|Al for hole only and ITO|LiF (1 nm)|***m*DPEPO** (100 nm)|LiF (1 nm)|Al for electron only, where MoO_3_ and LiF respectively served as hole- and electron-injecting layers, were fabricated to evaluate the intrinsic carrier transporting ability of ***m*DPEPO** (Fig. 5b). All of these materials show electron predominant characteristics with electron-only current density (*J*) remarkably larger than hole-only *J*. According to field-dependent space charge limited current model, the hole mobility (*μ*_h_) of **44′DPEPO** is estimated to be 1.20 × 10^–7^ cm^2^ V^–1^ s^–1^, which is 10 fold that of **24′DPEPO** and 100 fold those of **22′DPEPO** and **24DPEPO** (Table 1). On account of the **DPE**-localized HOMOs for ***m*DPEPO** and the electron-transporting character of DPPO, the hole transporting abilities of ***m*DPEPO** should be in direct proportion to the exposure degree of their **DPE** as the main hole transporting channel. Although the superposition of the two DPPOs renders the deepest LUMO that of **24DPEPO**, its electron mobility (*μ*_e_) is the lowest at 7.72 × 10^–9^ cm^2^ V^–1^ s^–1^, which is only one tenth of that of **22′DPEPO**. In contrast, the *μ*_e_ of **24′DPEPO** at 4.02 × 10^–6^ cm^2^ V^–1^ s^–1^ is dramatically improved by 3 orders of magnitude, which is almost equivalent to that of **44′DPEPO**. According to the DFT results, both **DPE** and DPPO are involved in unoccupied molecular orbitals and incorporated into electron transportation (Fig. S2†). In this case, the various *μ*_e_ of ***m*DPEPO** actually corresponds to their different intermolecular interplay. Compared to the over-concentration of DPPO with big steric hindrance in **24DPEPO** and **22′DPEPO**, by virtue of the relatively weaker steric effect of *para*-substituted DPPO and separation configurations, the intermolecular interplay of **24′DPEPO** and **44′DPEPO** is more effective to facilitate charge hopping between adjacent molecules. Furthermore, it is shown that the *J* of **24′DPEPO**-based devices is intermediate between those of **22′DPEPO** and **44′DPEPO**-based devices, revealing the carrier transporting ability of **24′DPEPO** as a combined result of steric and electronic coupling effects of its *ortho*- and *para*-substituted DPPOs. Nevertheless, on the contrary to **24DPEPO** with superposition configuration, the separation configuration of **24′DPEPO** causes the superiority of its *para*-DPPO in electrical performance enhancement.

In general, through an asymmetrical separation configuration incorporating both *ortho*- and *para*-DPPO substitutions, **24′DPEPO** successfully integrates the complementary advantages of **22′DPEPO** and **44′DPEPO** in favorable optical characteristics and improved electrical performance, respectively, revealing its great potential in exciton harvesting and utilization.

### 2.5. Device performance of deep-blue TADF diodes

**Fig. 6:**
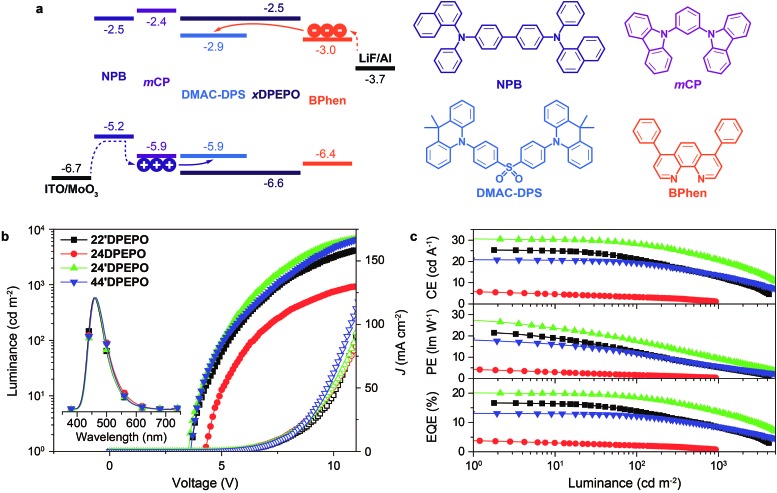
(a) Device configuration and energy level diagram of ***m*DPEPO**-based deep blue TADF diodes and the chemical structures of employed materials; (b) luminance–*J*–voltage curves and EL spectra (inset) of **DMAC-DPS**-based devices using ***m*DPEPO** as hosts; (c) efficiency *vs.* luminance curves of the deep blue TADF devices.

The well-controlled and differentiated optoelectronic properties of constitutional isomers ***m*DPEPO** establish the basis to selectively investigate the determinants of EL performance of blue TADF host materials. The devices were fabricated with a configuration of ITO|MoO_3_ (6 nm)|NPB (70 nm)|*m*CP (5 nm)|***m*DPEPO**:**DMAC-DPS** (20 nm, 10% wt)|***m*DPEPO** (5 nm)|Bphen (30 nm)|LiF (1 nm)|Al, in which MoO_3_ and LiF served as the hole and electron-injecting layer, NPB and Bphen are 4,4′-bis[*N*-(1-naphthyl)-*N*-phenylamino]biphenyl and 4,7-diphenyl-1,10-phenanthroline as hole and electron transporting layers and *m*CP (*N*,*N*′-dicarbazole-3,5-benzene) and ***m*DPEPO** were used as exciton-blocking layers, respectively (Fig. 6a). The doping concentration was optimized as 10% wt (Fig. S5†). In contrast to the large energy barriers of ∼0.5 eV between FMOs of ***m*DPEPO** and *m*CP and Bphen, the HOMO and LUMO energy levels of **DMAC-DPS** perfectly match with the corresponding FMO energy levels of *m*CP and Bphen, making the charge capture and exciton recombination on **DMAC-DPS** dominant in exciton harvesting. At high operation voltages, high-energy carriers can surmount the barriers to recombine on ***m*DPEPO**, making host-to-dopant energy transfer considerable. In this case, exciton quenching by host–host and host–dopant interactions can directly influence the device performance.

All of the devices showed deep blue emissions peaking at 460 nm at 1000 cd m^–2^ (inset in Fig. 6b). In comparison to the PL spectra, the hypochromatic shift of EL emissions should be attributed to the optical microcavity effect of nanometer-scaled devices. **22′DPEPO** and **24′DPEPO**-based devices achieved the highest color purity with Commission Internationale Ed I'eclairage (CIE) coordinates of (0.16, 0.17), owing to their rigid structures and LE-featured excited states (Table 2). In contrast, the relatively flexible structures and partially CT-type excited states of **24DPEPO** and **44′DPEPO** rendered broadened EL spectra with CIE coordinates of (0.16, 0.20) and (0.16, 0.18), respectively. The remarkably increased intensity of the long-wavelength part in the EL emission from **24DPEPO**-based devices was actually due to the decreased intensity of the short-wavelength part, which is readily quenched through nonradiative transitions during host–dopant interactions.

**Table 2:** EL performance of ***m*DPEPO**-based devices employing DMAC-DPS as the TADF emitter

Host	Operation voltage^*a*^ (V)	Maximum efficiency^*b*^	Efficiency roll-off^*c*^ (%)			Emission peak (nm)	CIE (*x*, *y*)
			CE	PE	EQE		
**22′DPEPO**	3.7, 5.4, 7.6	25.3, 21.5, 16.7	18, 50	44, 75	19, 50	460	0.16, 0.17
**24DPEPO**	4.3, 6.5, —	5.7, 4.2, 3.7	44, —	63, —	44, —	460	0.16, 0.20
**24′DPEPO**	3.5, 5.1, 7.0	30.6, 27.5, 20.1	8, 33	37, 66	8, 32	460	0.16, 0.17
**44′DPEPO**	3.6, 5.1, 7.5	20.8, 18.1, 13.1	7, 30	35, 53	7, 30	460	0.16, 0.18

^*a*^Operating voltages for onset, 100 and 1000 cd m^–2^.

^*b*^The maximum efficiencies of CE (cd A^–1^), PE (lm W^–1^) and EQE (%).

^*c*^At 100 and 1000 cd m^–2^.

*I*–*V* characteristics of these light-emitting devices showed a trend consistent with the charge mobility of their host materials (Fig. 6b). **44′DPEPO** endowed its devices with the highest *J*, while the *J* of **24′DPEPO**-based devices was secondary. Along with increasing operation voltage, the effect of **44′DPEPO** and **24′DPEPO** on enhancing *J* became more prominent. However, the difference between the *J* of these devices was remarkably smaller than that between the *J* of the single-carrier transporting devices, especially at low driving voltages, which indicated the significant contribution of **DMAC-DPS** to carrier injection and transportation. Nevertheless, **44′DPEPO** and **24′DPEPO** endowed their devices with a maximum luminance of ∼6500 cd m^–2^, which was more than 2000 cd m^–2^ higher than that of **22′DPEPO**-based devices; meanwhile, when using **24DPEPO** as a host, the maximum luminance was less than 1000 cd m^–2^. The higher luminance of **44′DPEPO** and **24′DPEPO**-based devices corresponded to higher exciton concentrations owing to larger and balanced charge flux in their EMLs. Furthermore, the driving voltages of **44′DPEPO**-based devices were 3.6, 5.1 and 7.5 V for onset, 100 and 1000 cd m^–2^, respectively, which were lower than those of **22′DPEPO**-based analogues (Table 2). **24′DPEPO** further reduced the driving voltages of its devices to 3.5, 5.1 and 7.0 V, which were about 1 V lower than those of **24DPEPO**-based devices. In comparison to **44′DPEPO** with stronger electroactivity, the lower driving voltages achieved by **24′DPEPO** actually reflected the higher exciton harvesting efficiency of its devices.^25^

On the basis of the four-layer device structure, **22′DPEPO**-based devices realized maximum efficiencies of 25.3 cd A^–1^ for current efficiency (CE), 21.5 lm W^–1^ for power efficiency (PE) and 16.7% for EQE, which were about 20% higher than those of **44′DPEPO**-based devices (Fig. 6c and Table 2). The superiority of **22′DPEPO** in the maximum efficiencies should be attributed to the effectively suppressed collision-induced quenching through reducing host–dopant interactions by virtue of the strong steric effect of its *ortho*-DPPOs. Significantly, in addition to the advantages of *ortho*-substituted DPPO, the unsymmetrical separation structure of **24′DPEPO** further weakens intermolecular interactions, improving the maximum efficiencies to 30.6 cd A^–1^, 27.5 lm W^–1^ and 20.1%, which are among the best results for deep-blue TADF devices reported so far.4a,5d,5e,5i,5j,16a Therefore, compared to **22′DPEPO**, the asymmetric configuration of **24′DPEPO** gives rise to the remarkable efficiency increase by 20%. In contrast, the maximum efficiencies of **24DPEPO**-based devices were the lowest among these devices, suffering from serious exciton quenching through structural relaxation-induced nonradiative transitions.

Despite larger maximum efficiencies, **22′DPEPO**-based devices showed serious efficiency reduction with roll-offs as large as 18 and 50% at 100 and 1000 cd m^–2^, respectively. With the best electrical performance among ***m*DPEPO**, **44′DPEPO** facilitated charge flux balance in the EML of its devices, enhanced exciton recombination and reduced redundant charge concentration, thereby decreasing exciton-polaron collision probability for TPQ suppression. Consequently, on the contrary to **22′DPEPO**, **44′DPEPO** supported its devices with remarkably reduced roll-offs as low as 7 and 30% at 100 and 1000 cd m^–2^, respectively. Significantly, **24′DPEPO**-based devices also realized low roll-offs of 8 and 32% at 100 and 1000 cd m^–2^, respectively, comparable to those of **44′DPEPO**-based analogues. The efficiency roll-offs of **24′DPEPO** and **44′DPEPO**-based devices are among the lowest values reported so far for deep-blue TADF diodes.4a,5d,5e,5i,5j,16a If proportional to their charge mobility, TPQ suppression in these devices can be roughly evaluated in the order **44′DPEPO** > **24′DPEPO** > **22′DPEPO** > **24DPEPO**. On the other hand, compared to the exposed T_1_ states of **22′DPEPO** and **44′DPEPO**, the extremely condensed T_1_ state of **24′DPEPO** can more effectively restrain collision-induced quenching, which offsets the slight inferiority of **24′DPEPO** to **44′DPEPO** in charge mobility. It would be rational that to some extent the enhanced efficiency stability at high luminance would reflect improved device durability, since device aging can be accelerated at high luminance to generate charge traps and exciton quenching sites, making both luminance and efficiency decrease.5e,^26^ In this case, **24′DPEPO** with reduced efficiency roll-off would be superior to **22′DPEPO** in device stability.

The state-of-the-art performance of **24′DPEPO**-based devices clearly shows that for high-energy-gap blue TADF host materials, the strong steric effect is beneficial to suppress TTA for high efficiencies; while, under high driving voltages, the improved charge mobility can facilitate flux balance in EML to suppress TPQ. Significantly, with an effectively protected T_1_ state with an extremely condensed location, the EL performance of asymmetric **24′DPEPO** was far beyond simple integration of those of **22′DPEPO** and **44′DPEPO**, verifying the great importance of suppressing host-involved intermolecular interactions. The sky-blue devices of another TADF dye 1,2-bis(carbazol-9-yl)-4,5-dicyanobenzene (**2CzPN**) employing **24′DPEPO** also showed higher efficiencies and reduced roll-off in contrast to **22′DPEPO**, displaying the universality of the host design strategy for **24′DPEPO** (Fig. S6†).

## 3. Experimental section

### 3.1. Materials and instruments

All the reagents and solvents used for the synthesis were purchased from Aldrich and Acros and used without further purification. **DPEPO** and **DPESPO** bromides were prepared according to our previous report.20*d*

^1^H NMR spectra were recorded using a Varian Mercury plus 400NB spectrometer relative to tetramethylsilane (TMS) as an internal standard. Molecular masses were determined using a FINNIGAN LCQ Electro-Spraying Ionization-Mass Spectrometer (ESI-MS), or a MALDI-TOF-MS. Elemental analyses were performed on a Vario EL III elemental analyzer. Absorption and photoluminescence (PL) emission spectra of the target compounds were measured using a SHIMADZU UV-3150 spectrophotometer and a SHIMADZU RF-5301PC spectrophotometer, respectively. Thermogravimetric analysis (TGA) and differential scanning calorimetry (DSC) were performed on Shimadzu DSC-60A and DTG-60A thermal analyzers under a nitrogen atmosphere at a heating rate of 10 °C min^–1^. Cyclic voltammetric (CV) studies were conducted using an Eco Chemie B. V. AUTOLAB potentiostat in a typical three-electrode cell with a platinum sheet working electrode, a platinum wire counter electrode, and a silver/silver nitrate (Ag/Ag^+^) reference electrode. All electrochemical experiments were carried out under a nitrogen atmosphere at room temperature in dichloromethane. Phosphorescence spectra were measured in dichloromethane using an Edinburgh FPLS 920 fluorescence spectrophotometer at 77 K cooled by liquid nitrogen with a delay of 300 μs using the Time-Correlated Single Photon Counting (TCSPC) method with a microsecond pulsed xenon light source for 10 μs to 10 s lifetime measurement, and a synchronization photomultiplier for signal collection and the Multi-Channel Scaling Mode of a PCS900 fast counter PC plug-in card for data processing. The absolute PLQY of the films was measured with an integrating sphere.

#### General phosphorylation procedure for bromides

**Scheme 1:**
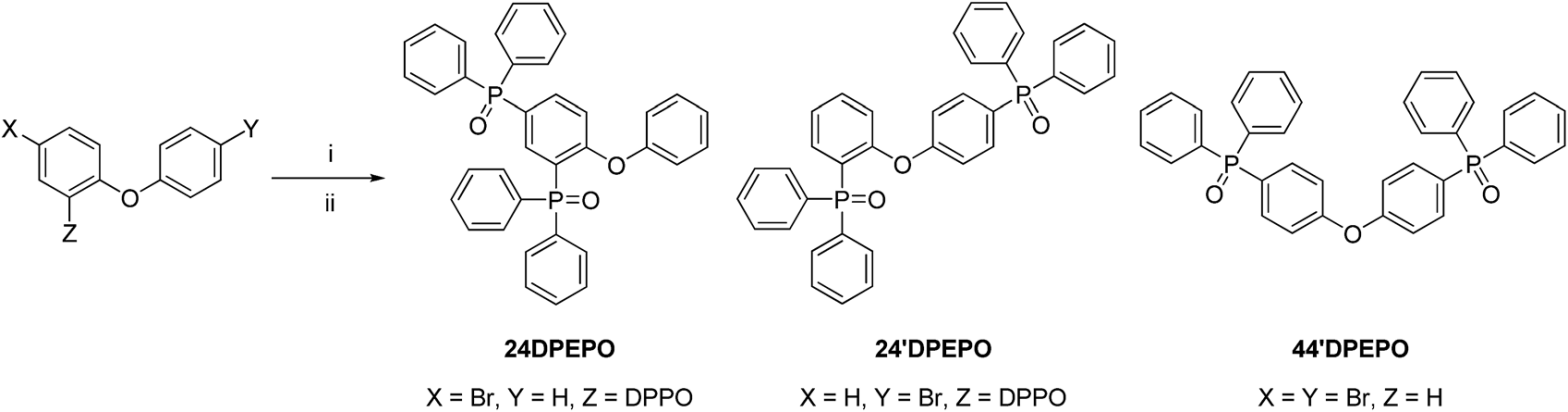
Synthetic procedure of **24DPEPO**, **24′DPEPO** and **44′DPEPO**. (i) Ph_2_PH, Pd(Ac)_2_, NaAc, DMF, 130 °C, 24 h; (ii) CH_2_Cl_2_, 30%H_2_O_2_, 0 °C.

In Ar_2_, bromide (1 mmol), NaAc (1.1 mmol), Pd(Ac)_2_ (0.05 mmol) and Ph_2_PH (1.1 mmol) were dissolved in DMF (10 mL) and heated to reflux for 24 h. After cooling to room temperature, water (10 mL) was added to the system, which was then extracted with CH_2_Cl_2_ (3 × 3 mL). The organic layer was combined and dried with anhydrous sodium sulfate. The solvent was removed *in vacuo* to afford the crude phosphine intermediate product. Then, the phosphine was dissolved in CH_2_Cl_2_ (10 mL). H_2_O_2_ (30%, 4 mL) was added to the solution dropwise and stirred for 2 h at 0 °C. Then, the mixture was extracted using CH_2_Cl_2_ (3 × 3 mL). The organic layer was combined and dried with anhydrous sodium sulfate. The solvent was removed *in vacuo*, and then the residue was purified by flash column chromatography to afford the phosphine oxide product (Scheme 1).

#### 2,4-Bis(diphenylphosphoryl)diphenylether (**24DPEPO**)

393 mg of white powder with a yield of 69%. ^1^H NMR (TMS, CDCl_3_, 400 MHz): *δ* = 8.009–7.957 (t, *J* = 10.4 Hz, 1H), 7.840–7.775 (t, *J* = 12.8 Hz, 1H), 7.717–7.606 (m, 8H), 7.566–7.284 (m, 14H), 6.862–6.821 (q, *J*_1_ = 4.4 Hz, *J*_2_ = 8.4 Hz, *J*_3_ = 1.2 Hz, 1H), 6.572–6.550 ppm (d, *J* = 10.0 Hz, 2H); LDI-TOF: *m*/*z* (%): 570 (100) [M^+^]; elemental analysis (%) for C_36_H_28_O_3_P_2_: C 75.78, H 4.95; found: C 75.82, H 4.97.

#### 2,4′-Bis(diphenylphosphoryl)diphenylether (**24′DPEPO**)

439 mg of white powder with a yield of 77%. ^1^H NMR (TMS, CDCl_3_, 400 MHz): *δ* = 8.062–8.007 (q, *J*_1_ = 7.6 Hz, *J*_2_ = 12.8 Hz, *J*_3_ = 1.6 Hz, 1H), 7.767–7.719 (q, *J*_1_ = 6.8 Hz, *J*_2_ = 12.4 Hz, *J*_3_ = 1.6 Hz, 4H), 7.667–7.616 (q, *J*_1_ = 7.2 Hz, *J*_2_ = 12.0 Hz, *J*_3_ = 1.2 Hz, 4H), 7.589–7.534 (q, *J*_1_ = 7.2 Hz, *J*_2_ = 14.8 Hz, *J*_3_ = 1.2 Hz, 3H), 7.506–7.315 (m, 3H), 6.930–6.897 (q, *J*_1_ = 5.2 Hz, *J*_2_ = 8.0 Hz, 1H), 6.665–6.638 ppm (dd, *J*_1_ = 2.0 Hz, *J*_2_ = 8.8 Hz, 2H); LDI-TOF: *m*/*z* (%): 570 (100) [M^+^]; elemental analysis (%) for C_36_H_28_O_3_P_2_: C 75.78, H 4.95; found: C 75.80, H 4.95.

#### 4,4-Bis(diphenylphosphoryl)diphenylether (**44′DPEPO**)

428 mg of white powder with a yield of 75%. ^1^H NMR (TMS, CDCl_3_, 400 MHz): *δ* = 7.691–7.621 (m, 12H), 7.563–7.523 (td, *J*_1_ = 1.2 Hz, *J*_2_ = 7.2 Hz, 4H), 7.482–7.438 (td, *J*_1_ = 2.8 Hz, *J*_2_ = 7.6 Hz, 8H), 7.105–7.078 (dd, *J*_1_ = 2.4 Hz, *J*_2_ = 8.8 Hz, 4H); LDI-TOF: *m*/*z* (%): 570 (100) [M^+^]; elemental analysis (%) for C_36_H_28_O_3_P_2_: C 75.78, H 4.95; found: C 75.77, H 4.94.

### 3.2. Density functional theory (DFT) and time-dependent DFT (TDDFT) calculations

DFT computations were carried out with different parameters for structure optimizations and vibration analyses. The ground states and triplet states of molecules in vacuum were optimized by the restricted and unrestricted formalism of Beck's three-parameter hybrid exchange functional^27^ and Lee, Yang and Parr’s correlation functional^28^ (B3LYP)/6-31+G(d,p), respectively. The fully optimized stationary points were further characterized by harmonic vibrational frequency analysis to ensure that real local minima had been found without imaginary vibrational frequency. The total energies were also corrected by zero-point energy both for the ground state and triplet state. Natural transition orbital (NTO) analysis was performed on the basis of optimized ground-state geometries at the level of (B3LYP)/6-31+G(d,p).^23^ The contours were visualized with Gaussview 5.0. All computations were performed using the Gaussian 09 package.^29^

### 3.3. Device fabrication and testing

Before loading into a deposition chamber, the ITO substrate was cleaned with detergents and deionized water, dried in an oven at 120 °C for 4 h, and treated with oxygen plasma for 3 min. Devices were fabricated by evaporating the organic layers at a rate of 0.1–0.2 nm s^–1^ onto the ITO substrate sequentially at a pressure below 4 × 10^–4^ Pa. Onto the electron-transporting layer, a layer of LiF with 1 nm thickness was deposited at a rate of 0.1 nm s^–1^ to improve electron injection. Finally, a 100 nm-thick layer of Al was deposited at a rate of 0.6 nm s^–1^ as the cathode. The emission area of the devices was 0.09 cm^2^ as determined by the overlap area of the anode and the cathode. After fabrication, the devices were immediately transferred to a glove box for encapsulation with glass cover slips using epoxy glue. The EL spectra and CIE coordinates were measured using a PR650 spectra colorimeter. The current–density–voltage and brightness–voltage curves of the devices were measured using a Keithley 4200 source meter and a calibrated silicon photodiode. All the measurements were carried out at room temperature under ambient conditions. For each structure, five devices were fabricated to confirm the performance repeatability. To make conclusions reliable, the data reported herein were the closest to the average results.

## 4. Conclusions

A series of **DPEPO**-type host materials named ***m*DPEPO** as constitutional isomers with two DPPO groups substituted on a **DPE** core at either the *ortho*- or *para*-position were designed and prepared. The steric effect of *ortho*-substituted DPPO and the electron coupling effect of *para*-substituted DPPO were successfully integrated through the separation configuration of **24′DPEPO**, giving rise to its harmonious excited-state characteristics and electrical performance inherited from **22′DPEPO** and **44′DPEPO**, respectively. The asymmetrical configuration and the dominant orientation effect of *ortho*-substituted DPPO on the T_1_ location extremely condense the T_1_ state of **24′DPEPO** onto a single phenyl, completely protected from intermolecular interactions. Consequently, **24′DPEPO** endowed its **DMAC-DPS**-based deep blue TADF diodes with state-of-the-art performance featuring high EQE beyond 20% and low roll-off at 32% at 1000 cd m^–2^, which was dramatically improved in comparison to those of **22′DPEPO** and **44′DPEPO** and successfully demonstrated its potential. This showed that besides common energy level optimization, more delicate and purposeful modulation on the optoelectronic properties of host materials is crucial for developing high-performance TADF diodes.

## Supplementary Material

Supplementary information
