# Supplementary material for: Extremely condensing triplet states of DPEPO-type hosts through constitutional isomerization for high-efficiency deep-blue thermally activated delayed fluorescence diodes
Source: Chem Sci. 2016 Jan 14;7(4):2870–82. doi: 10.1039/c5sc04848f (PMC6054027; doi:10.1039/c5sc04848f)
Supplement: Supplementary file 1 [file SC-007-C5SC04848F-s001.pdf]

Electronic Supplementary Material (ESI) for Chemical Science.
This journal is © The Royal Society of Chemistry 2016

Electronic Supplementary Information

Extremely Condensing Triplet States of DPEPO-Type Hosts through Constitutional Isomerization for High-Efficiency Deep-Blue Thermally Activated Delayed Fluorescence Diodes

Jing Zhang,‡ Dongxue Ding,‡ Ying Wei,* *Hui Xu**

Key Laboratory of Functional Inorganic Material Chemistry (Ministry of Education) & School of Chemistry and Materials, Heilongjiang University, 74 Xuefu Road, Harbin 150080, P. R. China

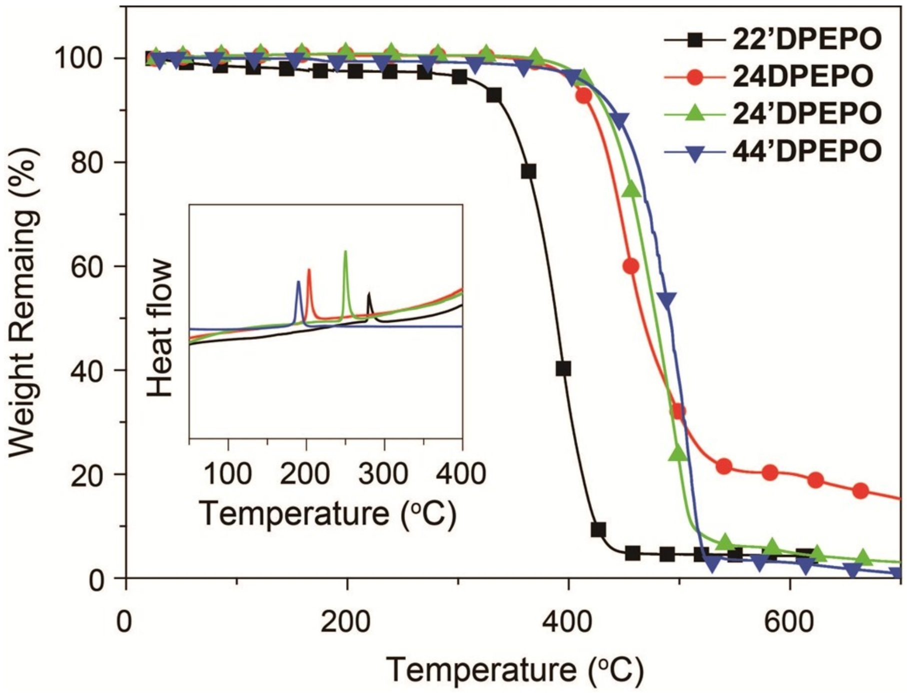

Figure S1. TGA and DSC curves of ***m*DPEPO**.

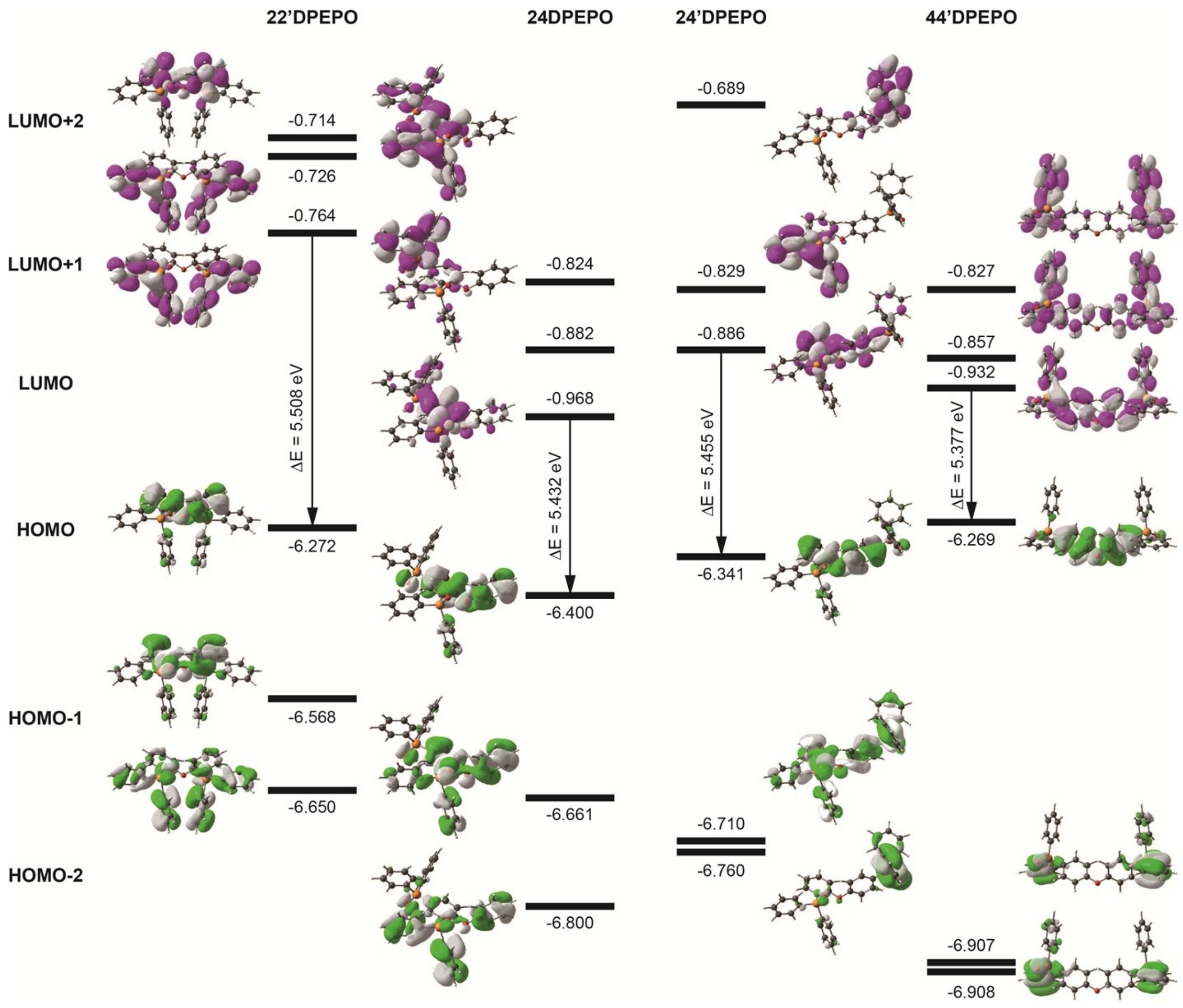

Figure S2. Energy levels and contours of FMOs of ***m*DPEPO**.

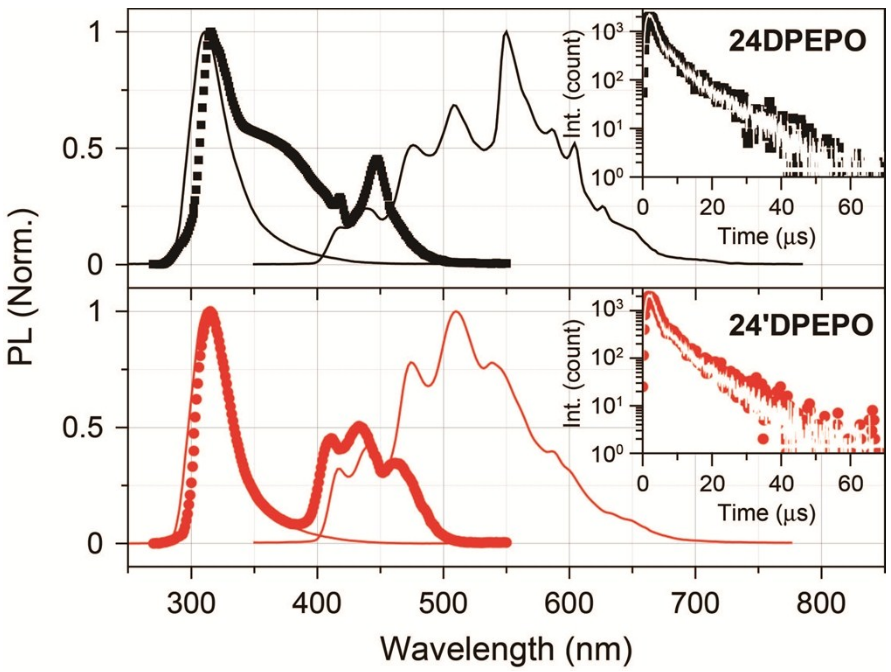

Figure S3. FL and PH in solution (line) and solid state emissions (symbol) of **24DPEPO** and **24'DPEPO**. Insets show the time decay curves of the 0-0 transitions from PH parts in solid state emissions (symbol) and PH spectra in solution (white line).

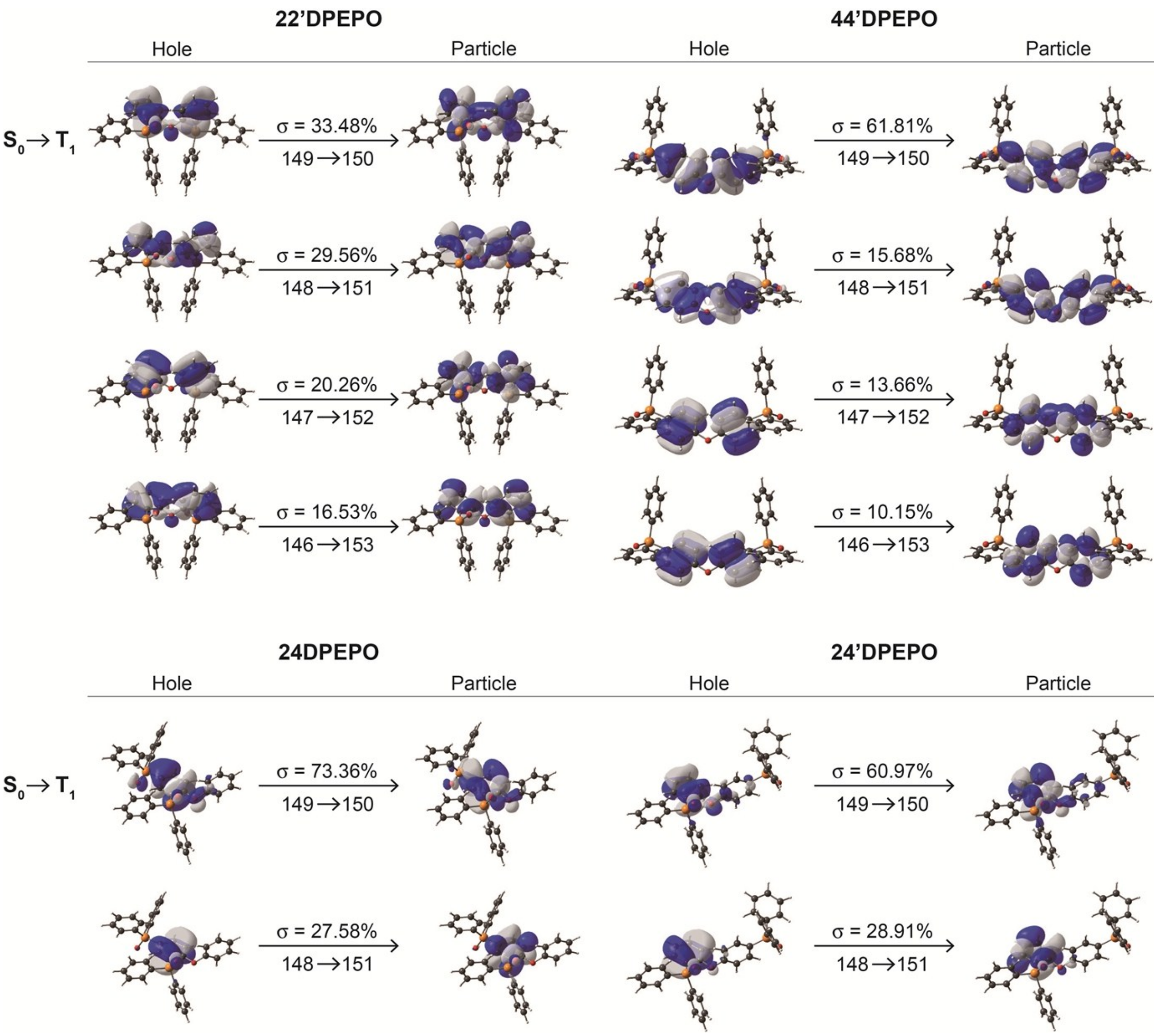

Figure S4. Major Natural transition orbitals (NTO) of S_0→T_1 states for ***m*DPEPO**. σ refers to the associated weight.

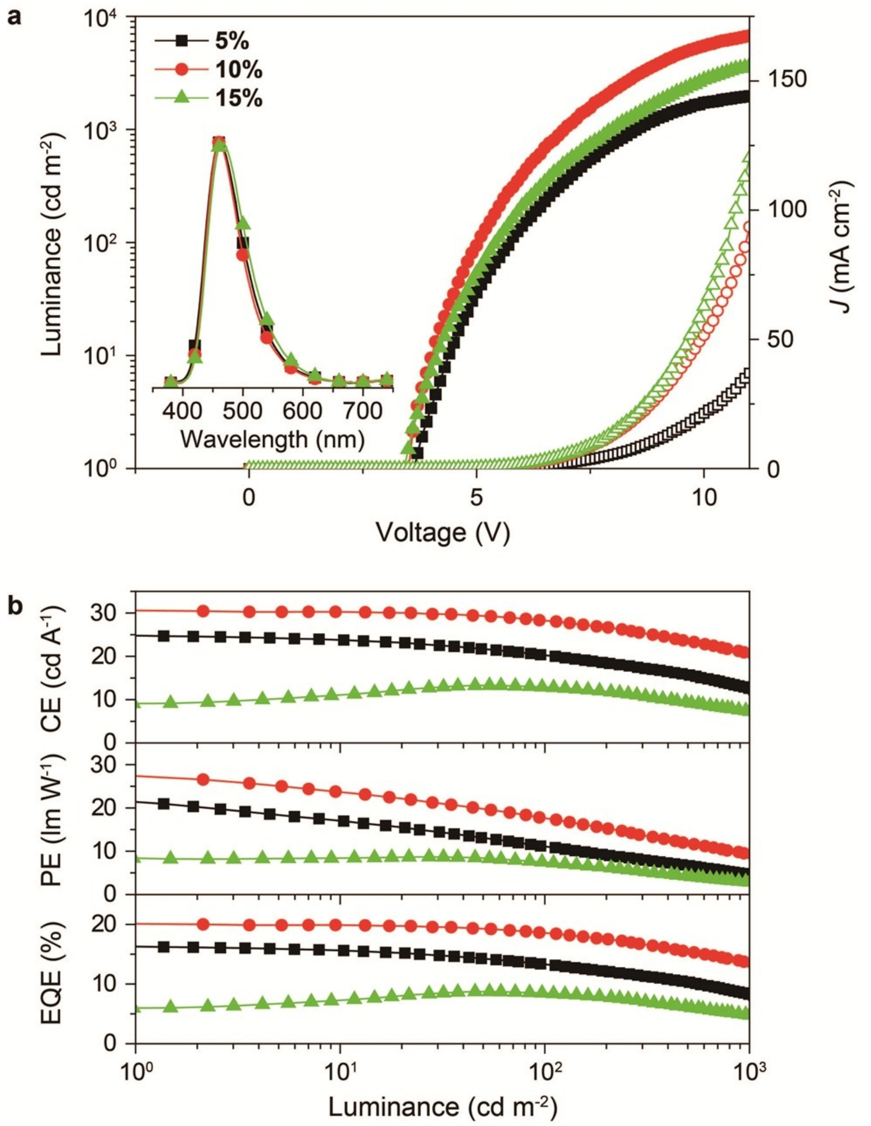

Figure S5. (a) Luminance-*J*-Voltage curves and EL spectra (inset) of **24'DPEPO**-based devices with **DMAC-DPS** doping concentrations of 5, 10 and 15%, respectively; (b) Efficiency *vs.* Luminance curves of the devices.

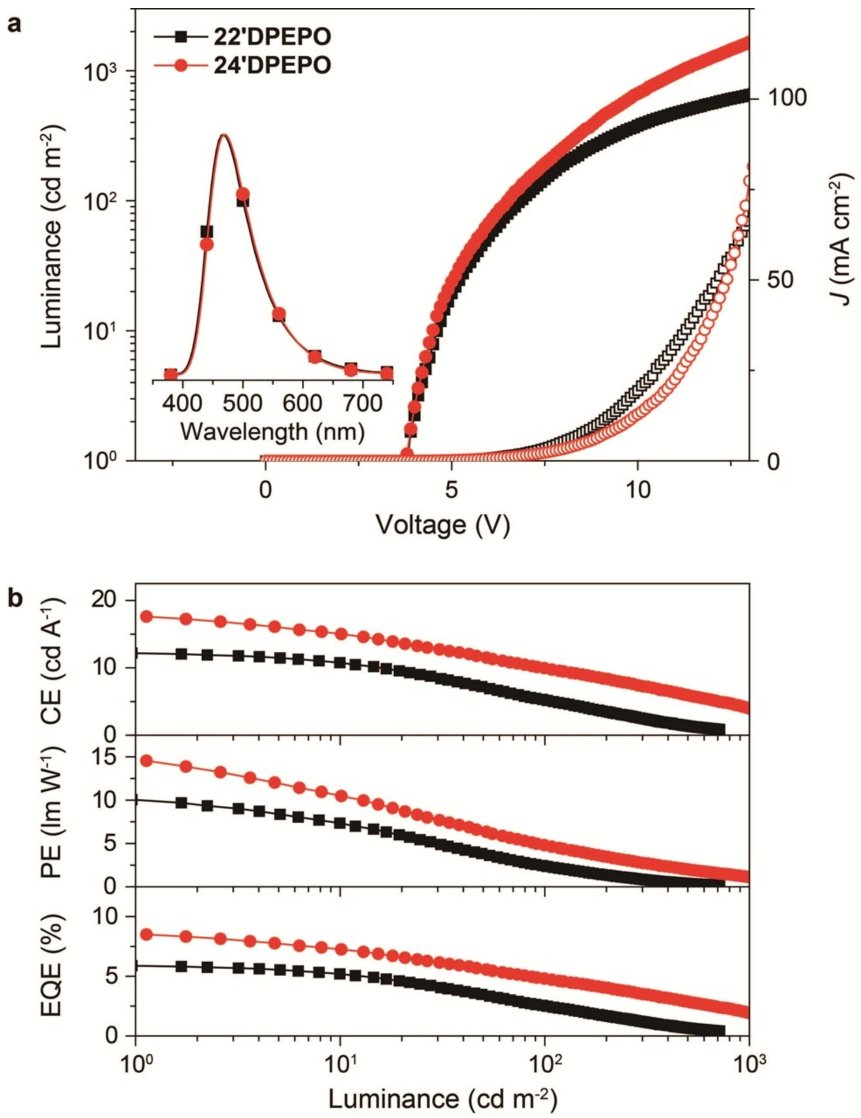

Figure S6. (a) Luminance-*J*-Voltage curves and EL spectra (inset) of **2CzPN**-based devices using **22'DPEPO** and **24'DPEPO** as host, respectively; (b) Efficiency *vs.* Luminance curves of the devices.
